# Preparation of Soft and Cohesive Gold for Matrix Work

**Published:** 1907-05

**Authors:** Wm. Crenshaw

**Affiliations:** Atlanta.


### PREPARATION OF SOFT AND COHESIVE GOLD FOR MATRIX WORK.

By Wm. Crenshaw, D. D. S., Atlanta.

Since the success of soft gold work depends materially on the form or preparation in which the gold is made up, I desire to offer in this paper a few brief suggestions with reference to this feature of the work.

Soft gold has had its advocates from time immemorial; and there have been two prominent preparations, and each of these with their advocates. The one consisting of moderate loose ropes or folded ribbon worked into place with what were called needle point pluggers. The other of cylinders, cushions and pelletts, worked with foot shaped instruments.

The first mentioned of these two preparations—the rope and the ribbons—have been made to do admirable work, particularly in the simpler forms of cavities, while the more difficult cavities such as occur between the bicuspids and molars in connection with improvised matrices, have employed cylinders, cushions and pellets.

To say we should use only one of these preparations would be unwise, and would stand in the way of the manipulating ability of some operators, because there are perhaps as many different ways—gradations so to speak—of manipulative gold and extend into those which resolve themselves into transition of method. Yet in the experience of the writer the results are more satisfactory to confine oneself to the cushion. With this preparation made rather denser than those of most operators, and used with foot-shaped pluggers, the cushion may be pushed unequivocally into position. The rope or ribbon is difficult to be properly condensed with needle points, if this be possible at all.

A misleading quality of the soft gold preparation is too great softness. By which I mean those preparations made so loose, down-like and rarified that the plugging instrument shall puncture and perforate rather than carry down the gold beneath the treading surface of the plugger, and so light, that when condensed it reduces into a bulk not large enough to bind in place in the larger forms of cavities. But when a proper amount of the foil is incorporated into the cylinder and this doubled and folded upon itself into a firm cushion so as to squeeze into the place under the pressure of the condensing instrument adapt to the walls of the cavity and hold in place, we have that form which facilitates and expedites our work.

The quality of non-cohesion is the soul of the soft gold. It is the stone, so to speak, which the builders have disallowed. Its non-cohesion is due in a measure to a structureless unit or crystal and is that quality which admits of the folds and surfaces doubled upon each other, sliding upon each other. And that quality which prevents cohesion of these surfaces, while the softness of cohesive gold is due to a perfect unit or crystal, and is that quality which from aggregated attraction causes union and welding of the molecule.

Soft gold can be condensed to a hurtful extent, i. e., over-condensed. And while we must value the quality of non-cohesion, we must realize at the same time that condensed to its ultimate density, we produce less than the best results, and tend to loosen our fillings rather than leave the joints hermetically sealed. Soft gold is therefore at its best when left at a point less than its greatest density. This may be true also of cohesive gold.

No one point eliminates so much of failure in the working of soft gold as to understand the value of firm preparations of the foil. The pioneers in soft gold work, Maynard, Badger Brothers, Emerson, who last lived and practiced at Macon in this State, and Phelps, still living at Columbus, and others, all used soft gold in very firm preparation, in the main in small compact cylinders.

The preparation best liked by the writer is to take a sheet of No. 4 soft gold foil and crinkle it; straighten this out, leaving it undulated; fold this three to four times, and twist into a loose rope, which may be cut into two or more pieces according to the size of the cavity and doubled twice, thrice or four times if necessary, and compressed into a wedge-shaped form, or into one which shall have thick center and compressed to thin edge around the periphery. (I shall be glad to demonstrate this to any interested during the clinic.)

The form next best liked is to take a large, loosely rolled cylinder and double it upon itself twice or oftener and bring it into a cushion the density of which is twice or three times as great as when we start with the cylinder, and consequenty twice to three times smaller.

One objection to working with cylinders is that if one becomes dependent on their nse, he is so inconvenienced, when out of the sizes needed, and again there is the objection of the useless investment of money in them.

One of the important advantages of the cushion preparation is that the operator is never at a loss for what he wants when foil is to be had; and he secures to himself a resource when he learns to make his preparations, which stands him in hand at all times. The matter of suiting the size of the cushion to the size of the cavity is a matter acquired in little experience.

The preparation of cohesive gold best liked by the writer is in the ribbon strip made by folding No. 4 twice upon itself, giving what is arbitrarily called No. 16. This is cut into strips suitable for the work in hand, and as long as kept clean and properly annealed, and the conbensed gold in which the operator is building, also kept clean, this preparation is reliable. Cohesive gold used in strips possesses one important feature not found in any other preparation of it, namely, its thinness, which enables the operator to carry it in between the teeth of close proximity and not to be divested of its cohesive property by pressure or partial condensation when compressed between teeth. None of the sponges, mosses, blocks, cylinders, or pellets admit of this to the same extent. Yet for finishing open surfaces, easily approached, a moss fibre, the sponge, light rarified Nos. ¼ to ½ cylinder, particularly if the cylinders are so condensed as to avoid doubling, lapping over and arching, are admirable for this purpose.

These latter mentioned preparations are susceptible of being made into the densest fillings, showing less of pits and taking on the highest polish.

